# Coronavirus Host Genetics South Africa (COHG-SA) database—a variant database for gene regions associated with SARS-CoV-2 outcomes

**DOI:** 10.1038/s41431-022-01089-8

**Published:** 2022-03-29

**Authors:** Fatima Barmania, Juanita Mellet, Megan A. Ryder, Graeme Ford, Candice L. Herd, Tsaone Tamuhla, Candice Hendricks, Rachel Giles, Thumbiko Kalua, Fourie Joubert, Nicki Tiffin, Michael S. Pepper

**Affiliations:** 1 Institute for Cellular and Molecular Medicine, Department of Immunology, SAMRC Extramural Unit for Stem Cell Research and Therapy, Faculty of Health Sciences, University of Pretoria, Pretoria, South Africa; 2 Centre for Bioinformatics and Computational Biology, Genomics Research Institute, Department of Biochemistry, Genetics and Microbiology, University of Pretoria, Pretoria, South Africa; 3 Computational Biology Division, Integrative Biomedical Sciences, Faculty of Health Sciences, University of Cape Town, Cape Town, South Africa; 4 Wellcome Centre for Infectious Disease Research in Africa, Institute of Infectious Disease and Molecular Medicine, University of Cape Town, Cape Town, South Africa

**Keywords:** Mutation, Genetic databases, Viral infection, Immunogenetics

## Abstract

The SARS-CoV-2 virus is responsible for the COVID-19 global public health emergency, and the disease it causes is highly variable in its clinical presentation. Clinical phenotypes are heterogeneous both in terms of presentation of symptoms in the host and response to therapy. Several studies and initiatives have been established to analyse and review host genetic epidemiology associated with COVID-19. Our research group curated these articles into a web-based database using the python application-server framework Django. The database provides a searchable research tool describing current literature surrounding COVID-19 host genetic factors associated with disease outcome. This paper describes the COHG-SA database and provides an overview of the analyses that can be derived from these data.

## Introduction

In December 2019, pneumonia cases of unknown aetiology were reported by local health facilities in Wuhan, the Capital of the Hubei province in China [1]. The causative coronavirus was subsequently identified and named severe acute respiratory syndrome coronavirus 2 (SARS-CoV-2) [2, 3]. The disease caused by the virus was later designated COVID-19 by the World Health Organization (WHO). One of the most striking features of COVID-19 is the remarkable heterogeneity in clinical presentation and patient outcomes. While some individuals remain entirely asymptomatic or present with mild symptoms, others progress to severe disease and death. Epidemiological studies have identified risk factors for severe disease, including older age, male sex, and comorbidities such as diabetes, hypertension, obesity, chronic respiratory diseases, cardiovascular disease, and cancer [4]. Some otherwise young healthy individuals, however, also succumb to COVID-19, and ongoing studies are now identifying host genetic makeup as an important contributor to disease outcome.

The importance of providing insight into the relationship between host genetics and susceptibility to SARS-CoV-2 infection and COVID-19 outcomes has prompted several initiatives. The COVID-19 Host Genetics Initiative (COVID-19 HGI) was established in early 2020 as a global resource with a shared purpose of generating genomic data that can be used to discover the genetic determinants of COVID-19 disease [5]. Their flagship genome-wide association paper reported 13 significant loci associated with susceptibility or severity and included two loci closely linked to a region on chromosome 3 that had been previously reported [6]. The discovery of this multigene locus, 3p.21.31, consisting of six genes and 22 variants was initially reported by Ellinghaus and co-workers [6]. It has since been replicated in other genome-wide association studies (GWAS) and is associated with a predisposition to increased risk of severe COVID-19 [7–10].

Another large initiative known as the Genetics of Mortality in Critical Care (GenOMICC) consortium, which contributes data to COVID-19 HGI, is a global collaboration that investigates the genetics of critical illness of various diseases and since 2020 has recruited individuals with severe forms of COVID-19. The team performed a GWAS analysis on 2 244 critically ill COVID-19 patients from Europe and identified and replicated three novel associations with COVID-19 found on chromosomes 19p13.3, 12q24.13, and 21q22.1 [8]. These loci represent five genes with key genetic differences found in critically ill patients compared to controls from population genetic studies in the United Kingdom.

Private genetics and biotechnology companies 23andMe, AncestryDNA and Regeneron have also launched COVID-19 GWAS studies related to susceptibility and disease severity. Data published using participant data in the 23andMe database demonstrated a strong association between blood group type and COVID-19 susceptibility as well as the replication of the chromosome 3p.21.31 locus for severity [7]. Roberts and colleagues used data from the AncestryDNA database and discovered the presence of three novel loci associated with COVID-19 implicated in viral replication and immunity [9] while Regeneron identified seven common genetic variants and three novel associations with COVID-19 possibly implicating a role for hyaluronan in COVID-19 severity [10].

The COVID Human Genetic Effort (COVID-19 HGE), another international consortium, is focused on identifying rare or common monogenic variations that underlie COVID-19 severity in previously healthy individuals or those variations that provide resistance to SARS-CoV-2 infection. Whole genome and exome sequencing done by this group revealed the presence of inborn errors in the Toll-like receptor 3 (*TLR3*) and interferon regulatory factor 7 (*IRF7*) genes, which affect interferon (*IFN*) type I responses [11].

Studies investigating the role of host genetics in COVID-19 outcomes are continually being published and thus the COHG-SA database was established to house study meta-data under one easily accessible resource. The COHG-SA database is envisioned as a searchable research tool to aid in refining future research, identifying parallel research efforts, and summarising the developing COVID-19 research landscape in the context of host genetic factors associated with susceptibility, severity, and patient outcomes. While it will provide a concise, consolidated resource for all researchers globally, it is also intended to assist COVID-19 researchers working with African participants and datasets. It is continually updated from available findings to augment the already comprehensive compilation of published genetic variants associated with COVID-19 susceptibility, severity, and outcomes. This paper describes the COHG-SA database and provides an overview of potential analyses that can be derived from these data.

## Methodology

**Fig. 1:**
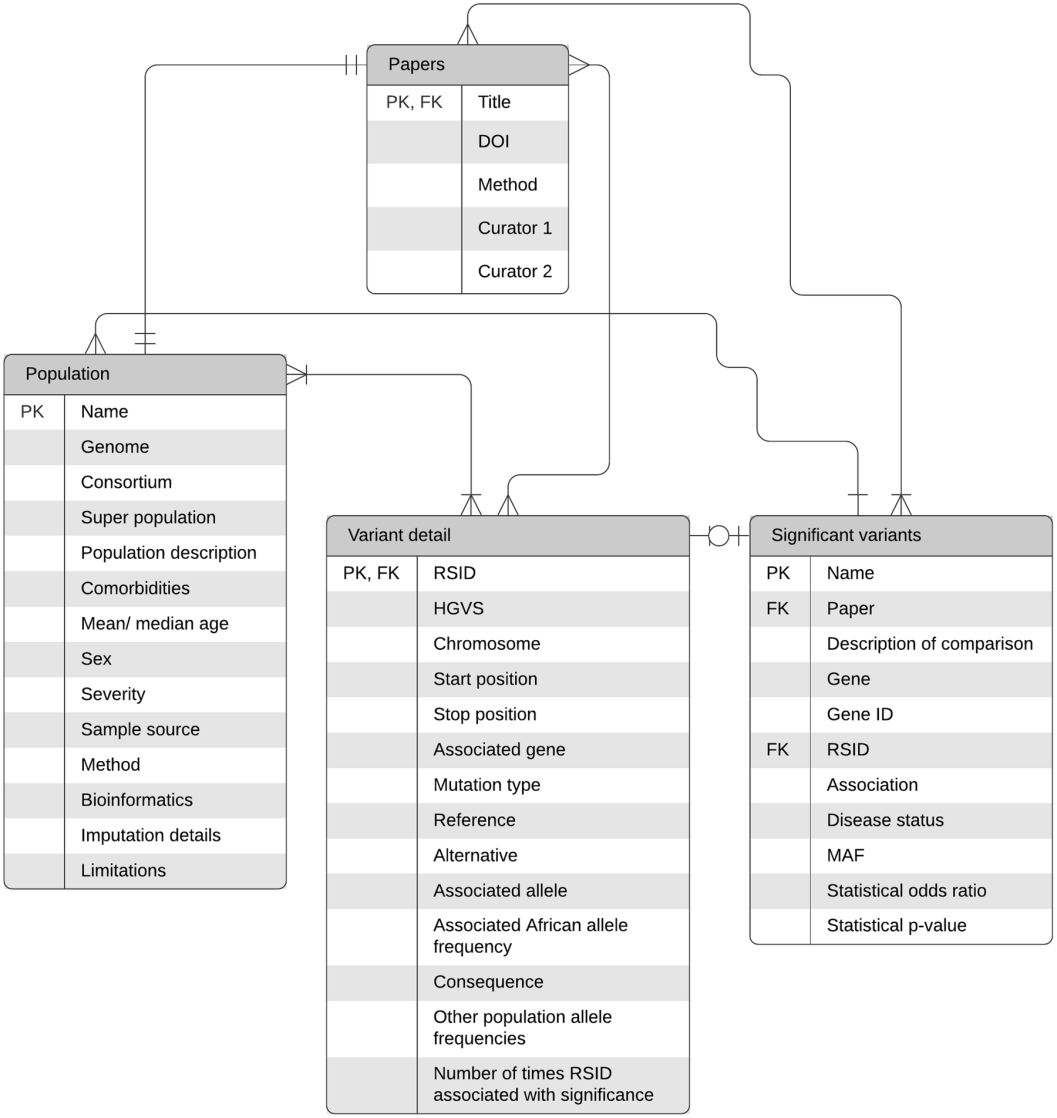
An entity-relationship model of the available information fields provided by the web interface. The four entities described in the database include Papers, Population, Variant detail, and Significant variants which each contain the associated attributes. The connecting lines showing the relationships of the entities are based on the engineering information style. The database column properties, glossary of terms, and interactors are found in Supplementary data A (Tables 1, 2, and 3). PK (Primary key), FK (Foreign key). Image created in Lucid Software Inc.

The COHG-SA database is a distillation of original COVID-19 host genetics research article data and demographics with useful external links and additional information at the fingertips of the user. Articles reporting on any genetic variants associated with COVID-19 disease susceptibility or severity outcomes in a COVID-19 affected cohort were included in the database. Exclusion criteria for studies included: (a) Those studies that only linked phenotypic data to COVID-19 outcomes and did not analyse any genetic data [12]; and (b) Studies reporting retrospective data of variants in genes associated with COVID-19, such as those involved in viral entry, but that did not analyse these variants in a particular COVID-19 affected cohort [13, 14]. Articles that meet the criteria are curated for inclusion in the COHG-SA database. Input data includes descriptions of study population demographics and summaries of the data where publications report statistically significant results, including a variety of additional fields to make the literature accessible and navigable (Fig. 1). Important metadata such as population origin, comorbidities, case and control population sizes and sample type as well as the outline of data acquisition methods have been captured from each paper. Significant variants, listed by reference SNP cluster ID (rsID), are tabulated with the allele frequency, comparison method, and statistical parameters from each paper. To provide context and facilitate cross-referencing, the associated gene(s), dbSNP page link, ENSEMBL gene ID, and association with disease status or susceptibility are provided for each significant variant. Variants can be individually explored or filtered by chromosome, nucleotide position, and variant consequence. Allele frequency in the test population can be compared to populations of interest using the NCBI Allele Frequency Aggregator (ALFA) resource link provided for each variant.

### Database set-up

The COHG-SA database (available at http://covgene.bi.up.ac.za) was compiled by South African researchers at the Universities of Pretoria and Cape Town, utilizing findings and data from papers posted or published between May 2020 and July 2021 (Table 1). Both peer-reviewed publications (13 of 18 entries) [5–8, 11, 15–22] and papers posted on the medRxiv preprint server (5 of 18 entries) [9, 10, 23–25] were reviewed to populate the database (Supplementary data A), recognising the unprecedented role of preprint servers in rapid dissemination of potentially critical COVID-19 research [26]. Article DOI’s are included for each database entry and are provided through hyperlinks in the user interface. This facilitates easy evaluation of the current status of the papers in terms of withdrawal or publication of preprints in journals and corrections or retractions for journal-published papers, given the rapid turnover of peer-reviewed publications and the absence of peer-review in preprints which could both affect the quality of the data [26–28]. A colour-coded pictogram can be found with the article title on the database to indicate if the article has been published or remains in pre-print. Each entry in the database has two appointed curators to monitor quality of input data - the first curator entering the data, and the second verifying the entries. A data freeze is performed monthly after assessing the literature and entering new publications as well as reviewing the status of those preprint publications already in the database. This database has been made available through a model-view-controller-based web interface, using the popular Python application-server framework Django, providing access from any internet-enabled device, with a mobile and desktop-accessible interface to facilitate dissemination and use across multiple sectors. The web interface has been structured as a series of SQL tables (Fig. 1) which can be accessed using a navigation menu. By making use of industry-standard mechanics such as dropdown menus (disease classifications, sample type, mutation type, and consequence), data standards are implemented and maintained by the curators, providing end-users with a standardised, robust resource suitable for scientific use.

**Table 1:** List of articles curated in the COHG-SA database at time of publication.

Date published	Title	DOI	Peer reviewed	Type of Publication	Consortium	Super-population	Method (s)	SARS-CoV-2 disease status	Significant Variant (s)	Source of genetic data
26/05/2020	APOE e4 Genotype Predicts Severe COVID-19 in the UK Biobank Community Cohort [12]	10.1093/gerona/glaa131	y	Letter to the editor	No	EUR	Genotype array	Severe	2	Retrospective UK Biobank
05/06/2020	ACE2 and TMPRSS2 variants and expression as candidates to sex and country differences in COVID-19 severity in Italy [13]	10.18632/aging.103415	y	Research paper	No	EUR	Microarray, WES, and GWAS	SARS-CoV-2 negative	none	none
17/06/2020	Genomewide association study of severe Covid-19 with respiratory failure [16]	10.1056/NEJMoa2020283	y	Research paper	The severe COVID-19 GWAS group	EUR	GWAS	Severe	2	Patients enroled in study
05/07/2020	Genetic risk factors for death with SARS-CoV-2 from the UK Biobank [21]	10.1101/2020.07.01.20144592	n	Research paper	No	EUR, SAS, AFR	GWAS	Critical	4	Retrospective UK Biobank
24/07/2020	Presence of Genetic Variants Among Young Men with Severe COVID-19 [18]	10.1001/jama.2020.13719	y	Preliminary communication	Radboud University Medical Center,Nijmegen, Netherlands	EUR, AFR	WES	Severe	1	Patients enroled in study
24/08/2020	Using symptom-based case predictions to identify host genetic factors that contribute to COVID-19 susceptibility [23]	10.1101/2020.08.21.20177246	n	Research paper	University of Groningen (Netherlands), EMBL, Vrije Universiteit Amsterdam, Amsterdam UMC, University of Helsinki, Broad Institute of MIT and Harvard, Massachusetts General Hospital, University of Edinburgh, Helix, University Medical Centre Utrecht	EUR, AMR	GWAS	Severe	5	Generation Scotland Biobank,Helix DNA Discovery project, Lifelines project & Netherlands Twin Register
27/08/2020	COVID-19 and Genetic Variants of Protein Involved in the SARS-CoV-2 Entry into the Host Cells [14]	10.3390/genes11091010	y	Research paper	No	EUR	WES	Asymptomatic, severe and critical	5	Patients enroled in study
09/10/2020	AncestryDNA COVID-19 Host Genetic Study Identifies Three Novel Loci [9]	10.1101/2020.10.06.20205864	n	Research paper	AncestryDNA	EUR, AMR	Meta-analysed GWAS	Severe & SARS-CoV-2 negative	5	Retrospective Ancestry DNA database
23/10/2020	Inborn errors of type I IFN immunity in patients with life-threatening COVID-19 [7]	10.1126/science.abd4570	y	Research paper	COVID Human Genetic Effort	Not specified	WGS & WES	Critical	9	Patients enroled in study
11/12/2020	Genetic mechanisms of critical illness in Covid-19 [6]	10.1038/s41586-020-03065-y	y	Research paper	GenOMICC, COVID-HGI, 23andme Inc	EUR	GWAS, microarray sequencing & WGS	Critical & severe	12	Patients enroled in study
11/12/2020	Host genetic effects in Pneumonia [17]	10.1016/j.ajhg.2020.12.010	y	Report	Vanderbilt University Medical Center biobank	EUR, AFR	GWAS	SARS-CoV-2 negative	1	Retrospective Vanderbilt University Medical Center biobank
16/12/2020	Common genetic variants identify therapeutic targets for COVID-19 and individuals at high risk of severe disease [10]	10.1101/2020.12.14.20248176	n	Research paper	Ancestry DNA, UK Biobank, Geisinger Health System, Penn Medicine Biobank	EUR, AFR, AMR, SAS	GWAS	Severe & SARS-CoV-2 negative	9	Retrospective Ancestry DNA, UK Biobank, Geisinger Health System, Penn Medicine Biobank
03/02/2021	Genetic variants are identified to increase risk of COVID-19 related mortality from UK Biobank data [15]	10.1186/s40246-021-00306-7	y	Research paper	No	EUR	GWAS	Severe	8	Retrospective UK Biobank
10/03/2021	Shared genetic aetiology between idiopathic pulmonary fibrosis and COVID-19 severity [20]	10.1016/j.ebiom.2021.103277	y	Research paper	Not specified	EUR	GWAS, Mendelian randomization	Severe	1	COVID-19 HGI
19/04/2021	Protective Role of a TMPRSS2 Variant on Severe COVID-19 Outcome in Young Males and Elderly Women [19]	10.3390/genes12040596	y	Communication	GEN-COVID Multicentre Study	EUR	WES	Severe	2	GEN-COVID Multicentre Study
22/04/2021	Trans-ancestry analysis reveals genetic and non-genetic associations with COVID-19 susceptibility and severity [8]	10.1038/s41588-021-00854-7	y	Research paper	23andMe COVID-19 team	EUR, AFR, AMR	GWAS	Severe & SARS-CoV-2 negative	2	23andMe database
18/05/2021	Japan COVID-19 Task Force: a nation-wide consortium to elucidate host genetics of COVID-19 pandemic in Japan [22]	10.1101/2021.05.17.21256513	n	Research paper	Japan COVID-19 Task force	EAS	GWAS	Severe	9	Patients in the study
08/07/2021	Mapping the human genetic architecture of COVID-19 [5]	10.1038/s41586-021-03767-x	y	Research article	COVID-19 Host Genetics Initiative	MID,S/EAS,AFR,AMR,EU	GWAS	Severe	13	COVID-19 Host Genetics Initiative

### Database functionality

Search functionality has been included in the web interface, allowing for the searching of results by title term, variants, variant details, position as well as by rsID. The filter and rank options can be used to rank entries by gene symbol (gene name), disease status, minor allele frequency (MAF), odds ratio (OR), and *p*-value, and further filtered by paper title, gene symbol, disease status, minimum MAF, minimum OR, and minimum *p*-value. This provides users with a more comprehensive filter and rank functionality and allows more complex queries to be performed. In addition to this, the Filter and Rank window contains a pre-set search button for (a) ranked gene list, which returns the entries ranked alphabetically by gene names; and (b) ranked variants list, which returns entries sorted by the number of entries that report each variant as having a significant effect on COVID-19 susceptibility, severity, or outcome.

We used the filter and rank tool to demonstrate the end usage of the database by analysing a list of 124 genes that were ranked by the gene symbol option and were identified in the articles included in the database as being significant in COVID-19 disease outcomes (Supplementary data B, Table 1). Various web-based tools could be used for pathway analysis to obtain a broad view of the types of pathways and cellular mechanisms enriched for in this gene list. Two web-based tools were used to showcase how one could identify and functionally annotate the genes and identify key gene clusters, pathways, and interacting proteins that are enriched in the list of genes found thus far to be associated with COVID-19 disease. The web-based tool DAVID (Database for Annotation, Visualization, and Integrated Discovery; http://david.abcc.ncifcrf.gov; version 6.8) [29] was used to identify enrichment of Gene Ontology (GO) terms from the biological process (BP), cellular component (CC), and molecular function (MF) ontologies [30, 31]. The protein-protein interactions (PPI) of the encoded proteins of genes associated with COVID-19 from the COHG-SA database were analysed using STRINGdb (Search Tool for the Retrieval of Interacting Genes/Proteins; version 11.0) [32]. Further information regarding the methodology for GO analysis using these tools can be found in the supplementary data (Supplementary Notes).

## Results

### Database overview

#### Papers curated in the database

The database contains data from 18 articles, of which 13 have been published in peer-reviewed journals (Table 1). As of 31 July 2021, five of the papers in the database were available as preprints in medRxiv (Table 1). Many of the studies (17/18) identified one or more variants that had a statistically significant association with SARS-CoV-2 susceptibility and severity. Only one paper [16] did not have variants that were included in the significant variant section of the database (Supplementary data A). This study was an exploratory study that did not use data from COVID-19 affected individuals. The variants indicated in this paper that possibly affect COVID-19 outcome, however, were included in the variant section of the database. Articles that identified variants that did not reach significance were also added to the variant section. Statistically significant variants were included in the significant variant section according to the parameters and *p*-values defined by the paper itself. If the variant was found to be statistically significant and replicated in more than one paper or study group, it would be reflected as a value in the last column of the variant details page.

Most of these publications were collaborative outputs from consortia that included the COVID-19 HGI, COVID-19 HGE, GenOMICC and the Japan COVID-19 Taskforce and others, detailed in the population table (Supplementary data A). While six studies collected patient samples prospectively to generate genetic data [6, 8, 11, 17, 20, 24], 12/18 used readily available retrospective genetic data curated at various national and institutional biobanks and commercial biotechnology companies such as 23andMe. This demonstrates the importance of existing sample and data infrastructure in being able to conduct rapid and responsive research.

#### Population demographics

The European (EUR) superpopulation was the most represented group in all the papers except for one study which had a population of only East Asian (EAS) participants [24]. The only African-ancestry population represented in the studies were African Americans, with no data from participants originating from the African continent (Table 1). This emphasizes the need for increasing representation of African populations in COVID-19 host genetic studies. Most studies focused on finding variants impacting COVID-19 disease severity, generally defined into two categories: (a) hospitalization for COVID-19 symptoms or; (b) critically ill requiring hospitalization and respiratory support or death due to COVID-19. Studies that included susceptibility analysis based their definition on a positive SARS-CoV-2 lab test, clinician confirmed COVID-19 or self-reported COVID-19 via questionnaires with or without symptoms. Most of the articles (13/18) in the database used GWAS to identify variants. Of the remaining five studies three used whole exome sequencing (WES), one used a genotype array and one study used both WES and whole genome sequencing (WGS) (Table 1).

#### Common genetic loci

Table 2 shows the genetic loci replicated in more than one article in the database. Each locus can be tagged by a number of variants such as the *LZTFL1*/3p21.31 locus which is composed of eight different tagged variants from seven papers included in the database (Supplementary A). This region encompasses many genes and may indicate a multi-trait locus. The *ABO* gene locus was also found in six different research studies and is associated with an effect on COVID-19 susceptibility. The *IFNAR2* and *DPP9* genes were indicated in four and three studies respectively while *TYK2*/*ICAM*, *TMEM65*, *TMPRSS2*, *OAS* gene family, *FOXP4*, and *CCHCR1* genes were indicated in two studies.

**Table 2:** Number of studies replicating various genetic loci.

Genetic loci/Official gene symbol	Number of research studies replicating gene region
*LZTFL1* (3p.21)	7
*ABO*	6
*IFNAR2*	4
*DPP9*	3
*TYK2*, *ICAM*	2
*TMEM65*	2
*TMPRSS2*	2
*OAS* gene family	2
*FOXP4*	2
*CCHCR1* (MHC)	2

### Use case for the database: gene ontology analysis

We have provided this use case as an example of the type of analysis that can be done using the data from this database. A comprehensive GO analysis was performed on the gene list created by the filter and rank function on the COHG-SA database, using external web-based tools.

Key findings from the DAVID analysis indicate that several genes (13/124) were mainly associated with the known biology of the interferon (IFN) response to viral infections (GO:0060337; GO:0060333; GO:00051607) (Supplementary Data B, Table 6). Many of the genes in the analysed list fall into the category of processes involved in immunity and antiviral defence.

STRINGdb analysis was used to generate a PPI network (Supplementary Notes, Supplementary Fig. 1) utilizing the genes from the database as input with 20 additional protein interactors in both the first and second shells of the network. The network consisted of 33 nodes and 127 edges, of which 27 nodes formed the main connected component of the network. The PPI network was significantly enriched (Supplementary Data C, Table 11) for ontology terms relating to the type I interferon pathway (*n* = 19), cytokine signalling in immunity (*n* = 14), innate immunity (*n* = 13), regulation of the immune response (*n* = 19) and the JAK-STAT signalling pathway (*n* = 8). A table containing the complete results from this case study analysis, with functional and pathway enrichment terms associated with the interacting proteins can be found in Supplementary C, Table 12.

## Discussion

The COHG-SA database is a platform containing published data from which potential genetic determinants of COVID-19 disease have been collated and can be analysed. COVID-19, however, remains a complex disease, and outcomes are unlikely to result from a single causative gene but rather multiple genes and environmental factors which co-contribute to the different phenotypes identified. Genetic loci that were replicated significantly in more than one paper are highlighted in Table 2 of the results section and information identifying these or other variants of interest can be accessed easily by filtering out variants in the database.

The 3p21.31 locus represents several independent signals that were previously associated with severity. However, Roberts and co-workers as well as the COVID-19-HGI consortium illustrate that this locus may also be involved in COVID-19 susceptibility [5, 9]. Further investigation will be required to ascertain if there are any missing causal variants within this region since none of the lead variants have been associated with other disease traits [5].

The ABO locus was also identified in more than one study. Studies have indicated that blood group O is associated with a protective effect on susceptibility whereas blood group A is associated with increased susceptibility to infection [12, 13]. The *IFNAR2*, *DPP9* and various other genes indicated in Table 2 have also been identified in more than one paper. The association of a variant of interest with disease status as well as its statistical significance and allele frequencies for each study can be easily accessed using the database.

The replicated variants and or loci should be included in further investigations to establish the mechanism by which these genes impact on the pathogenesis of COVID-19 disease since they have been identified by multiple study groups and, recognising that most loci were found by GWAS implies that the causal gene still needs to be identified. Results should be interpreted with caution as significance may be a result of the statistical power of the study, as was indicated by analysis of the data we have collated in the database to date, which suggests that some genes or loci were found to be significant in some studies but not in others.

To illustrate potential types of onward analysis of data from this database, we have demonstrated how significant variants identified may be linked to known gene functions and pathways which might underlie disease risk. The GO data analysis identifies key molecular functions based on the gene list in the database which may play a role in determining COVID-19 outcomes. Specifically, the IFN pathway appears to be implicated through many of the identified variants indicated in the database articles, as identified previously in the innate immune response to SARS-CoV-2 [11, 20, 33], and IFN therapy has already been used in clinical trials for COVID-19 patients who present with severe disease and is currently an approved therapeutic drug.

GO analysis of the significant variants and genes in the COHG-SA database also identified an enrichment of genes with cytokine and chemokine functions, reflecting previous studies that showed elevated levels of cytokines resulting in the cytokine release syndrome in patients with severe COVID-19 [34].

Research into the association of genetics and COVID-19 will continue beyond the current pandemic. This database facilitates the housing of these publications and associated meta-data which can then be mined efficiently by researchers for downstream use. The GO analysis is intended to demonstrate just one possible end usage.

The unique and extensive within-population diversity in African populations, arising from the earliest origins of modern humans in Africa, is well-documented [35]. Despite this, the genes and variants identified in the papers included in the COHG-SA database focus on populations of European ancestry with minor inclusions of Asian population groups and even less of African American populations. The major genetic locus, 3p21.31, replicated in seven papers, including the paper published by Namkoong and co-workers [24] using data from the Asian population, is associated with increasing risk for COVID-19 severity and has been shown to be inherited from Neanderthals [36]. The Neanderthal haplotypes are almost completely absent in African populations but are found at an allele frequency of 30% in South Asia and 8% in Europe. The GenOMICC consortium, in addition to the risk loci on chromosome 3, also identified seven other loci on chromosome 6, 12, 19, and 21 [8]. One of these loci, a haplotype on chromosome 12, is associated with a protective effect as it can reduce COVID-19 severity. This locus has also been shown to be inherited from Neanderthals [37]. Data from African individuals are clearly lacking in COVID-19 genomic research, even though analyses have suggested that African populations are disproportionately affected by severe disease. The disparities found in COVID-19 cases in various ethnic groups have been shown in data from health records in different countries, but whether these differences are due to socioeconomic or genetic factors is still unclear [38, 39]. The COHG-SA database includes the allele frequencies for the African population group derived from the allele frequency aggregator, ALFA, for each variant curated in the database. Considering the lack of COVID-19 host genetic data in African populations, the inclusion of the African ALFA allele frequency will be useful in determining which alleles should be prioritized in future studies in these populations.

## Future directions, gaps, conclusion

The COHG-SA database provides a platform of curated publications and study meta-data and findings, which can be used as a basis for exploring the genetic effect of COVID-19 disease. The collated data enables the identification of knowledge gaps in currently published research and can encourage investigation in potential areas of importance in disease. Considering the impact of COVID-19, databases like this one will assist the scientific community to increase collaboration efforts by easily and quickly accessing current data under one curated source. It is hoped that future studies done on African populations will be added to this resource and will benefit African COVID-19 research initiatives that aim to understand the effects of host genetics on outcomes in African patients. Although a significant number of genetic loci were indicated in the curated papers, many of these loci were determined using GWAS and therefore the causative variants and genes are still unknown. Going forward, how can one best use this information? We would first need to determine which of the identified candidate genes have an association with an effect size that indicates a true increased risk. These genes could potentially be incorporated into a genetic risk score for each patient. Patients identified to be high risk could then have treatment escalated early, or as novel treatments develop, be given targeted therapies to mitigate these risks. These strategies are, however, a long way off, needing validation studies and clinical trials before they can be implemented. For African populations, we await at least some data to guide our decision-making on a genomic tool for COVID-19 risk prediction, as previous experience has shown that extrapolating from present knowledge may not be sufficiently informative. We would also need to determine whether such an intervention is affordable and thus feasible in a resource-constrained country and continent.

Future plans for the database include integrating data analysis tools for gene prioritization as well as tools to create graphs and visual aids which will further increase the utility of the database to researchers.

## Disclaimer

Many of the papers recorded in the database were published early in the pandemic. Thus, many of these associated genes still need to be replicated or confirmed with their link to COVID-19. Additionally, certain studies reviewed in this database are preprints and have not yet been peer-reviewed. As such they should not be used as a guide for clinical or therapeutic purposes.

## Supplementary information

Supplementary Notes and Supplementary Figure 1

Supplementary Data A

Supplementary Data B

Supplementary Data C

## Data Availability

All data generated or analysed during this study are included in this published article, supplementary information files, and the database repository. Access to the repository is available at (http://covgene.bi.up.ac.za).
