# Supplementary material for: Coronavirus Host Genetics South Africa (COHG-SA) database—a variant database for gene regions associated with SARS-CoV-2 outcomes
Source: Eur J Hum Genet. 2022 Mar 29;30(8):880–8. doi: 10.1038/s41431-022-01089-8 (PMC8960680; doi:10.1038/s41431-022-01089-8)
Supplement: Supplementary file 1 — Supplementary Notes and Supplementary Figure 1 [file 41431_2022_1089_MOESM1_ESM.pdf]

Supplementary Notes:

Methodology used for DAVID analysis:

Genes associated significantly with COVID-19 outcomes from the various genomic studies were entered as a gene list and annotations were considered statistically significant with a Benjamini adjusted significance score (False discovery rate- FDR) of ≤0.05 (1). In addition, the DAVID functional annotation clustering function was used to classify the genes into the different biological pathway clusters based on the Kyoto Encyclopedia of Genes and Genomes (KEGG) and Reactome reference databases (FDR≤0.05). DAVID was also used to identify which of these genes have prior associations to complex diseases in the Genetic Association Database (GAD), and which types of diseases are enriched in these associations using a fold enrichment and associated FDR (FDR≤0.05).

Methodology used for STRINGdb analysis:

The STRING database contains information on over 24 million proteins from 5 090 organisms and can be used to depict known protein interactions from curated databases. It also identifies experimentally determined findings and protein interaction predictions using gene neighbourhood algorithms, gene co-expression, fusions and co-occurrence, protein homology, and text mining. Functional enrichment information on GO functions and processes, Universal Protein Resource (UniProt) terms and KEGG or Reactome pathways of the interacting proteins, can also be imported through the database. In the network, proteins are displayed as nodes while edges are used to represent the known or predicted relationships between proteins. For the purposes of this study, proteins encoded by genes in which we had listed variants involved in COVID-19 severity or susceptibility were used as input and species were restricted to Homo Sapiens. The PPI network was created with a high level of confidence (score ≥ 0.7) including a maximum of 10 additional protein interactors in the first and second shells, respectively.

Text-mining interaction sources, which provide information on the protein interactions from abstracts of scientific literature, were excluded from the analysis due to the higher likelihood of false positive predictions. Cytoscape software v3.8.2 was used to visualise and add functional enrichment information to the PPI network.

References

1. Benjamini Y, Hochberg Y. Controlling The False Discovery Rate - A Practical And Powerful Approach To Multiple Testing. J Royal Statist Soc, Series B. 1995;57:289-300.

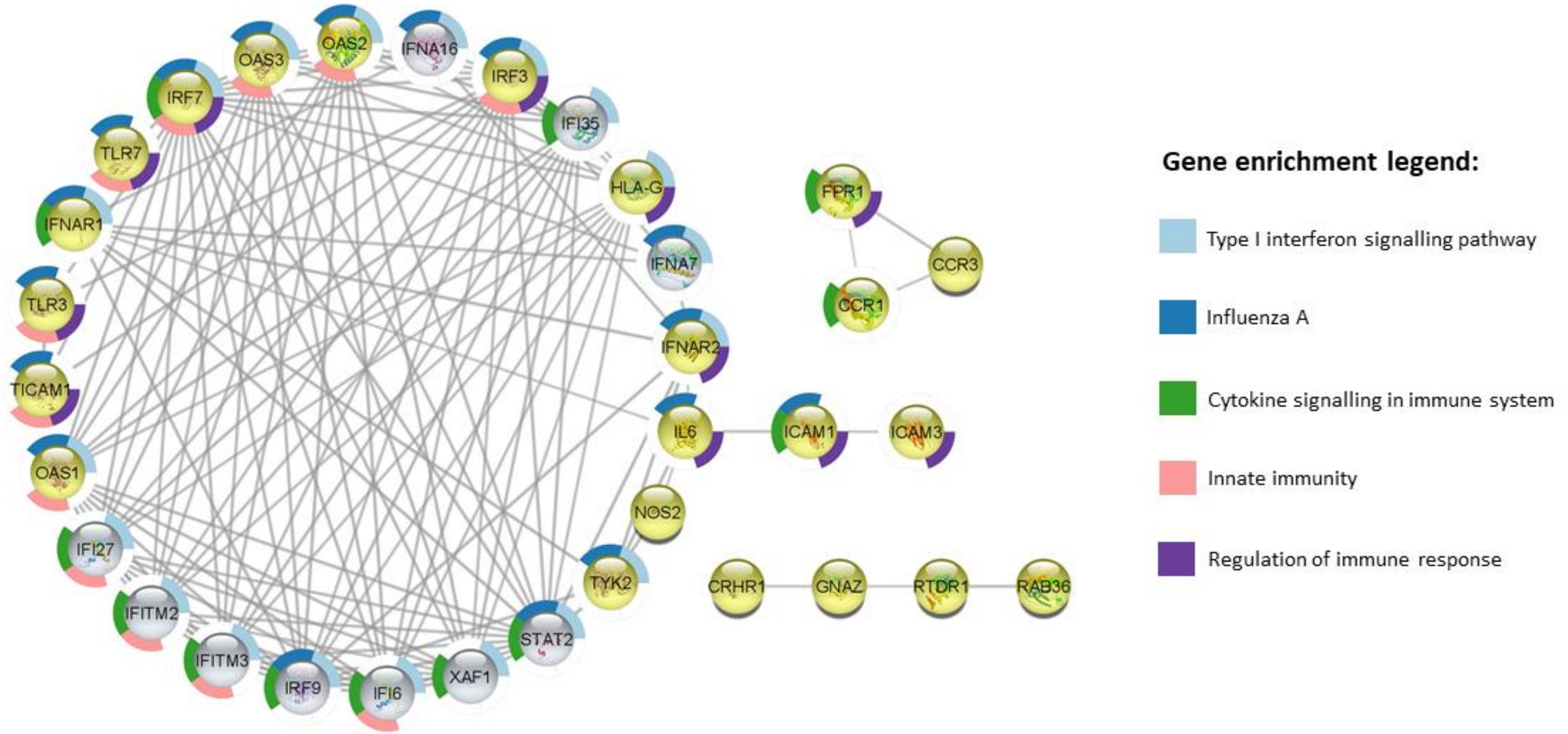

Supplementary Figure 1: Protein-protein interaction network describing the known and predicted interactions of proteins of genes associated with COVID-19 susceptibility or severity from the COHG-SA database. Yellow nodes are representative of the input set of proteins, while additional direct interactors of these proteins are shown in grey. Nodes are annotated with coloured borders (colour codes shown in key) representing significantly enriched GO terms or pathways.
